# Relationship between demographic and social variables and performance in virtual reality among healthcare personnel: an observational study

**DOI:** 10.1186/s12909-024-05180-0

**Published:** 2024-03-04

**Authors:** Daniel Katz, Benjamin Hyers, Eric Patten, Darren Sarte, Mariano Loo, Garrett W. Burnett

**Affiliations:** 1 Department of Anesthesiology, Pain, & Perioperative Medicine, Icahn School of Medicine at Mount Sinai, 1468 Madison Avenue, 10029 New York, NY USA; 2 Department of Academic Learning Environment, Ross University School of Medicine, 1600 SW 80th Terrace, Suite106A, 33324 Plantation, FL USA

**Keywords:** Virtual reality, Mixed reality, Adult learners, Medical education

## Abstract

**Background:**

Virtual reality is emerging as an important component of medical education. Although the benefits of virtual reality are apparent, the optimal strategy to orient to or differentiate learners in the virtual space have not been delineated. The purpose of this study was to investigate the relationships between demographic variables, social variables, and self-perceived comfort with technology to performance on a standardized non-medical virtual reality experience.

**Methods:**

This observational study was performed at the International Meeting on Simulation in Healthcare in 2022. This conference includes medical and non-medical attendees. Participants provided demographic information and participated in a scored non-medical VR experience due to the heterogeneity of the sample. Participants then completed a System Usability Index and NASA Task Load Index form. Participants were dividedintolow scoring, medium scoring, and high scoring groups according to their final game score for further analysis.

**Results:**

95 participants were included in final analysis. 55 (57.9%) of participants had prior virtual reality experience. Higher scores were associated with younger age (11.09, *p* < 0.001), identifying as male (11.09, *p* < 0.001), and a higher frequency of playing video games in the past (18.96, *p* < 0.001). The high score group was more likely to report comfort with virtual reality (6.29, *p* = 0.003) as well as comfort with new technology (4.61, *p* = 0.012). NASA Task Load Index scores trended down and System Usability Index scores trended up with increasing score. Being a nurse was a positive predictor of a higher score when compared to physicians in the multivariate analysis.

**Conclusion:**

Performance during an immersive virtual reality experience was most closely related to age, gender, and frequency of playing video games. Self-perceived comfort with virtual reality was more predictive of score than prior virtual reality experience.

**Supplementary Information:**

The online version contains supplementary material available at 10.1186/s12909-024-05180-0.

## Introduction

Virtual Reality (VR) has emerged as a popular teaching modality throughout medical education, where it has served to supplement and/or replace traditional teaching modalities, including high fidelity simulation (HFS) [1–3]. Other benefits of VR when compared to HFS include a high level of immersion associated with head-mounted VR, lower resource utilization including facilities, equipment, and staff, lower costs, and more flexibility in location and repeatability of training modules [1, 4]. While VR may provide benefits to medical educators, the innovative technology does have potential pitfalls. The novelty of VR could lead to resistance in adopting the technology by users with no prior experience [5, 6]. This may be due to it being an unfamiliar platform or lack of confidence in navigating the new technology. Given the cost of VR, experiences outside of the education space have been limited to enthusiasts only, which has limited uptake. This has changed significantly in the last two years with the advent of all-inclusive stand-alone hardware with lower entry costs, but VR is still not considered “mainstream” [7]. Finally, VR sickness, or motion sickness associated with VR use, may limit the utilization of VR as this is a known adverse effect of immersive VR [8, 9].

As utilization increases, educators and developers must consider how best to orient learners of all types not only to the educational experience, but to VR itself. Current literature linking demographic variables, prior technological experience, and scored-based outcomes is lacking. Furthermore, there is no current literature on the connection between prior technology and VR experience or self-perceived level of technology proficiency and success in VR environments. Finally, most prior works focused on one specific medical or surgical discipline per study, limiting generalizability [3, 4, 10]. Therefore, the purpose of this study was to investigate the connections between demographic variables such as age and gender, social variables such as level of education and prior use of videogames, along with self-perceived comfort with technology to performance on a standardized non-medical VR experience.

## Methods

### Study design

This study was performed in line with the principles of the Declaration of Helsinki and institutional review board approval was granted through the Mount Sinai Program for the Protection of Human Subjects Institutional Review Board (STUDY-21-01752). Written informed consent was obtained from all participants. The datasets used and analyzed during the current study are available from the corresponding author on reasonable request.

Participants attending the International Meeting on Simulation in Healthcare (San Diego CA USA) who attended the Technology Experience Area (TEA) were invited to participate. Meeting attendees share a common interest in simulation in healthcare and occupations represented include simulation administrators, operations specialists, engineers (hardware, software, biomedical), emergency medical technicians, nurses, nurse practitioners, physician assistants, respiratory therapists, physicians and military personnel. Attendees with medical backgrounds also come from a variety of specialties including but not limited to emergency medicine, trauma surgery, general surgery, urology, anesthesiology, internal medicine (and subspecialties) and others, making it one of the most diverse healthcare conferences. A convenience sample size was used given the research setting for this observational study. Advertisements for the TEA session were found in the conference handbook and announcements on social media were made prior to each of the sessions. The TEA was offered on 3 different conference days for two sessions each day, one from 9am to 12pm and one from 1pm to 4pm. Prior registration for this experience was not required, and all subjects were “walk-ins”. Only adult subjects (> 18 years old) were recruited to participate in this study.

After obtaining informed written consent, participants completed a demographic survey including information about their prior experiences with technology. This included information regarding age, gender, race/ethnicity, occupation, highest level of education, and prior experiences with technology and virtual reality using both multiple-choice questions and VAS 0-100 mm scales calculated to the nearest millimeter. They were then escorted to a VR station. Each station was a 10ft x 10ft square box with an Oculus Quest 2 (Meta Technologies, Menlo Park CA USA) headset, 2 hand-held VR controllers, and a haptic vest (Bhaptics, Daejeon South Korea). The hardware was preset with the Synth Riders (Kluge Interactive, Los Angeles CA USA) game. This is a rhythm-based experience whereby participants are asked to touch color-coded orbs, which correspond to musical notes, using one or both hands. With each correct note, the controllers and haptic vest provided haptic feedback through vibrations. TEA staff proctored the session only to aid participants in getting into and out of the headset as well as ensure that the area remained clear of any obstructions. TEA staff were instructed not to answer questions about the experience or provide hints to participants as to how to play the game. Participants were not offered a tutorial on the software. The same song (“Carol of the Bells Remix”) on the easy setting was used. This song was selected as it is a commonly recognizable song to the general public and was a relatively short experience (under 3 min). This non-medical game was selected due to the wide variety of occupations and knowledge base of potential participants at this conference, which allowed for a standardized assessment of success in virtual reality. At the end of the session, the final game score was recorded by the proctor and participants then completed a System Usability Index and NASA-Task Load Index form (NASA-TLX). These previously validated, publicly available tools test 10 domains on a scale of 50 and 6 domains on a scale of 600 respectively. A low final game score was determined as 0-100,000, a medium final game score as 100,001 to 300,000, and a high final game score was any score over 300,000. The thresholds for these groups were agreed upon by the investigators prior to data analysis based on pilot data.

### Statistical analysis

Statistical analysis was performed on SPSS® IBM (Aramonk NY USA) Version 24. All continuous variables were tested for normality via Shapiro-Wilk test as well as visual inspection of distributions. Normal distributed variables are reported as mean (SD) and non-normal distributions as median [IQR]. Differences and impacts for demographic variables are reported as F statistics and *p*-values. A *p*-value of <0.05 was deemed significant. Categorical comparisons were performed with Chi-Square or Fischer’s Exact test as needed. Participants were placed into 3 groups based on their scores (low, medium, high). Statistics involving three groups where multiple comparisons were performed Bonferroni correction was applied. Analysis of associations between score and other variables were performed via linear regression. Due to the exponential nature of the scoring system, a natural log transformation was performed on the score data to create a normal distribution of scores to fulfil the requirements for linear regression. As such, output coefficients from the regression analysis should be interpreted as proportional changes. Finally, multivariate regression was performed with all variables included as they were deemed by the researchers as potential confounders. Groups with less than 10 subjects were combined when applicable to stabilize the model. Co-linearity was examined with an a priori threshold of variance inflation factor (VIF) of 5.0 as determining co-linearity. Co-linearity statistics can be seen in Supplemental Table 1.

## Results

### Participant characteristics

A total of 97 participants participated in the study, 2 of which were eliminated from the data set due to missing scores as well as large portions of the surveys missing (> 70%), leaving 95 participants for analysis. Demographic information as well as prior technology and VR experiences are displayed in Table 1, which is stratified according to low, medium, and high scores. Of note most participants reported prior experiences with VR prior to participation in the study 55 (57.9%) and reported a modest degree of comfort in VR (60 [21.75-86]) based on a 0-100 mm VAS scale, however the variation was large. The most popular prior VR headset used was the Quest platform (1 or 2) for which 41 (74.5%) of participants with prior experience had utilized. This was then followed by the HTC platform 17 (30.9%), Windows Mixed Reality headsets 7 (12.7%), the Valve Index 6 (10.9), and the HP Reverb (G1 or G2) 5 (9.1%). 6 (10.9%) participants were unsure which headset was used in their prior experience. Prior usage of video games in the past was also highly variable (47.7%-78.3%). Of the 26 participants who reported never playing videogames the most common reason was that video games did not exist when they were younger 14 (53.8%). This was followed by a general aversion to video games 7 (26.9%), no financial resources to play video games 3 (11.5%), and parental disallowance of video games 1 (3.8%).

**Table 1:** Demographic and prior technology and virtual reality experiences

Variable	All Participants (*n* = 95)	Low Scoring (*n* = 44)	Medium Scoring (*n* = 28)	High Scoring (*n* = 23)	F Statistic (*p*-value)
**Age**	44 [36–55]	51.5 [42.25–60.75]	40.5 [35-52.75]	37 [32–41]	11.09 (< 0.001)
**Gender**					13.38
Male	54 (56.8)	15 (34.1)	17 (60.7)	22 (95.7)	(< 0.001)
Female	40 (42.1)	29 (65.9)	10 (35.7)	1 (4.3)	
Non-binary	1 (1.1)		1 (3.6)	0 (0)	
**Race**					0.10
American Indian	1 (1.1)	1 (2.3)	0 (0)	0 (0)	(0.901)
Asian Black Pacific Islander White Other Declined	16 (16.8) 8 (8.4) 1 (1.1) 62 (65.3) 5 (5.3) 2 (2.1)	6 (13.6) 5 (11.4) 1 (2.3) 27 (61.4) 2 (4.5) 2 (4.5)	4 (14.3) 3 (10.7) 0 (0) 19 (67.9) 2 (7.1) 0 (0)	6 (26.1) 0 (0) 0 (0) 16 (69.6) 1 (4.3) 0 (0)	
**Occupation**					0.29
Physician APP (PA/NP) Nurse Other Healthcare Sim Operations Other	21 (22) 5 (5.3) 23 (24.2) 3 (3.2) 14 (14.7) 29 (30.5)	5 (11.4) 5 (11.4) 15 (34.1) 2 (4.5) 5 (11.4) 12 (27.3)	7 (25.0) 0 (0) 5 (17.9) 1 (3.6) 5 (17.9) 10 (35.7)	9 (39.1) 0 (0) 3 (13.0) 0 (0) 4 (17.4) 7 (30.4)	(0.746)
**Highest Degree**					1.36
Graduate Undergraduate High School	62 (65.3) 26 (27.4) 7 (7.4)	29 (65.9) 14 (31.8) 1 (2.3)	20 (71.4) 6 (21.4) 2 (7.1)	13 (56.5) 6 (26.1) 4 (17.4)	(0.261)
**Prior VR Use**					2.97
Yes	55 (57.9)	21 (47.7)	16 (57.1)	18 (78.3)	(0.055)
**Play Video Games**					18.96
**Growing Up**					(< 0.001)
Daily Weekly Monthly Rarely Never	18 (18.9) 26 (27.4) 10 (10.5) 15 (15.8) 26 (27.4)	3 (6.8) 9 (20.5) 6 (13.6) 7 (15.9) 19 (43.2)	2 (7.1) 10 (35.7) 3 (10.7) 6 (21.4) 7 (25.0)	13 (56.5) 7 (30.4) 1 (4.3) 2 (8.7) 0 (0)	
**Comfort VR**	60 [21.75-86]	41 [14-77.5]	65 [25–95]	82 [53–92]	6.29 (0.003)
**Comfort New Tech**	80 [57.75-91]	75 [47-84.75]	80 [66–100]	84 [75–100]	4.61 (0.012)
**Comfort Smart Phone**	93 [83.75–100]	90 [81.25–100]	94 [84–100]	96 [86–100]	0.64 (0.530)
**Comfort Computer**	93 [86.5–100]	93 [82.5–100]	94 [87–100]	93 [87–100]	0.319 (0.728)
**Use New Tech Read Manual**					5.46 (0.006)
Always Most of the time Some of the time Rarely Never	10 (10.5) 19 (20.0) 39 (41.1) 23 (24.2) 3 (3.2)	4 (9.1) 8 (18.2) 21 (47.7) 7 (15.9) 3 (6.8)	5 (17.9) 8 (28.6) 12 (42.9) 3 (10.7) 0 (0)	1 (4.3) 3 (13.0) 6 (26.1) 13 (56.5) 0 (0)	
**Use New Tech Tutorial**					1.60 (0.207)
Always Most of the time Some of the time Rarely Never	7 (7.4) 21 (22.1) 47 (49.5) 17 (17.9) 2 (2.1)	3 (6.8) 11 (25.0) 17 (38.6) 10 (22.7) 2 (4.5)	4 (14.3) 5 (17.9) 17 (60.7) 2 (7.1) 0 (0)	0 (0) 5 (21.7) 13 (56.5) 5 (21.7) (0)	

APP: Advanced Practice Provider, PA: Physician Assistant, NP: Nurse Practitioner

### Analysis of performance

**Fig. 1:**
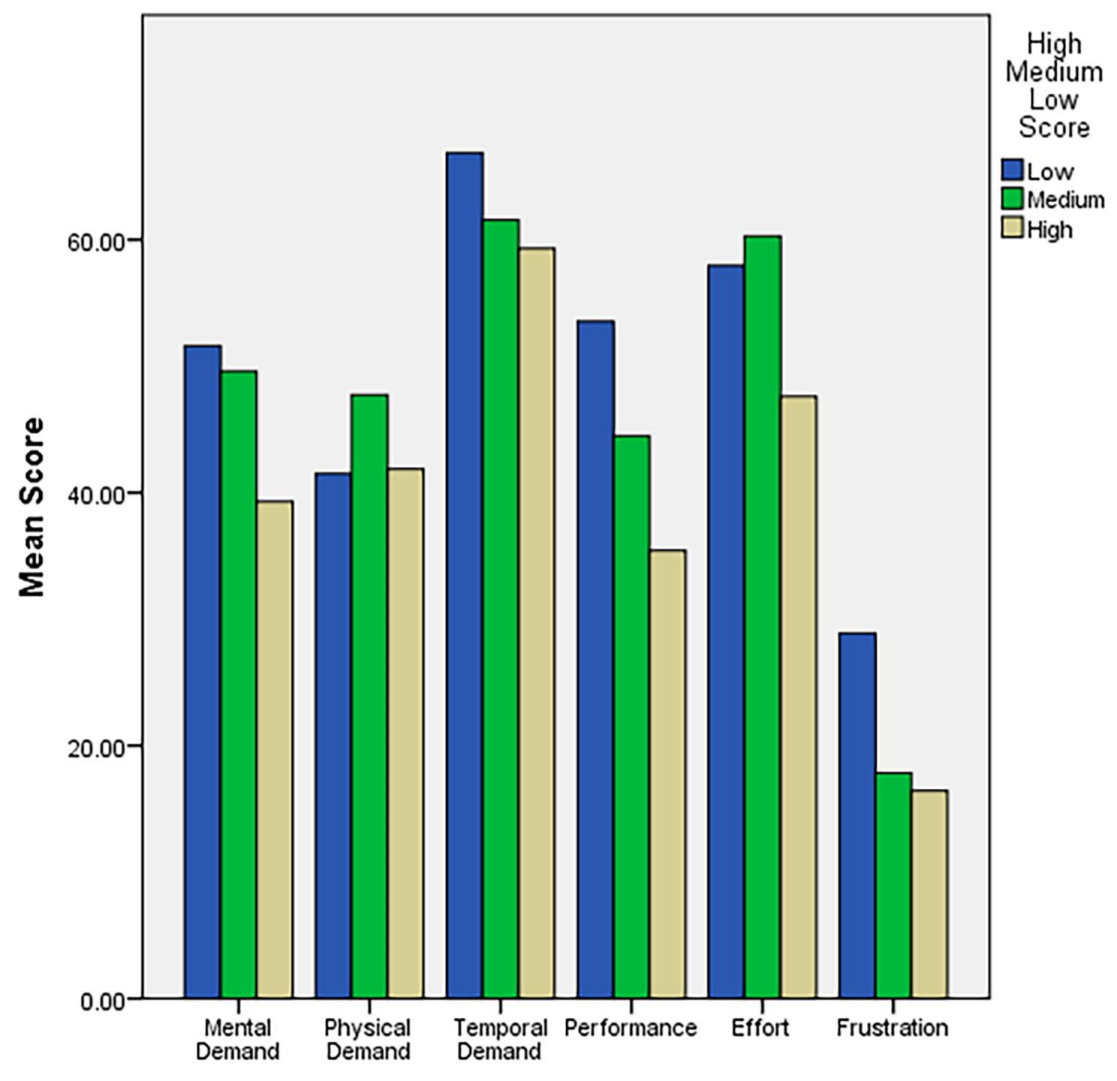
NASA-TLX scores for each domain stratified by low, medium, and high scores

Stratifying participants by score category revealed some significant associations. Higher scores were associated with younger age (11.09, *p* < 0.001), identifying as male (11.09, *p* < 0.001), and a higher frequency of playing video games in the past (18.96, *p* < 0.001). The high score group was also more likely to report comfort with VR (6.29, *p* = 0.003) as well as comfort with new technology (4.61, *p* = 0.012) and were less likely to read instruction manuals when confronted with new technology (5.46, *p* = 0.006). Score values as well as System Usability and NASA-TLX values are presented in Table 2. In general, usability scores were high albeit higher in the higher scoring groups. This was also mirrored in the NASA-TLX scores which trended down as user score increased. NASA-TLX scores separated by item are seen in Fig. 1.

**Table 2:** System usability and NASA-TLX values

Variable	All Participants (*n* = 95)	Low Scoring (*n* = 44)	Medium Scoring (*n* = 28)	High Scoring (*n* = 23)	*p* value
**Score**	108,130 [58,350 − 293,142]	56,290 [35,528-76999]	140,990 [114,756 − 216,574]	435,789 [344,096–679,797]	-------
**System Usability Index Total Score** (50)	45 [40–47]	42.5 [35–46]	46 [41-47.75]	46 [45–48]	0.007
**Nasa-TLX Total Score** (600)	283 [2131 − 352]	311.5 [240–369]	291.5 [211–354]	238 [175–336]	0.045

**Fig. 2:**
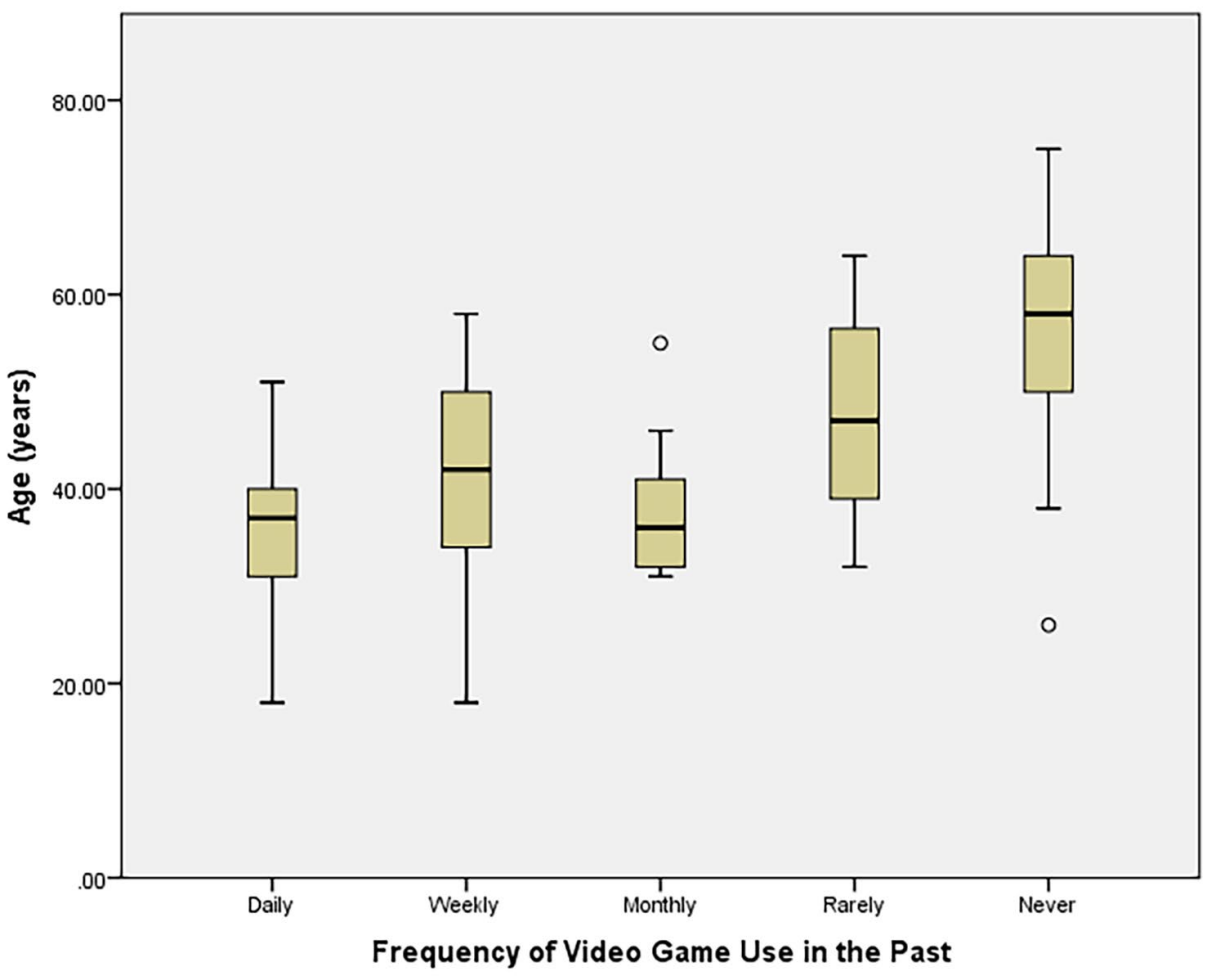
Age of subjects stratified by frequency of video game use in the past (*p* < 0.001)

Univariate analysis via linear regression is found in Table 3. The strongest predictors of score were age (-0.031 [-0.046 - -0.017], *p* < 0.001) and gender (-1.081 [-1.41 - -0.747], *p* < 0.001). Occupation and prior use of VR were also significant independent predictors. Prior use of video games, either daily or weekly, were associated with higher scores (Fig. 2) as was self-reported comfort in VR or with new technology as well as comfort level with using a television.

**Table 3:** Univariate analysis via linear regression

Univariate Variable	Coefficient	95% CI	*p*-value
**Age** (years)	-0.031	-0.046- -0.017	< 0.001
**Gender** (male)			
Female Non-binary	-1.081 -0.551	-1.41- -0.747 -2.13- 1.030	< 0.001 0.494
**Race** (Caucasian)			
Asian African American Other	0.206 -0.496 -0.460	-0.311–0.722 -1.187–0.195 -1.151–0.230	0.436 0.159 0.191
**Occupation** (Physician)			
Nurse Other Healthcare Sim Operations Other Non-Healthcare	-0.840 -1.101 -0.285 -0.343	-1.388 - -0.292 -1.838 - -0.364 -0.915–0.344 -0.859–0.173	0.003 0.003 0.374 0.193
**Highest Degree** (Graduate)			
Undergraduate/HS	0.168	-0.241–0.576	0.421
**Prior Use of VR** (Yes)			
No	-0.547	-0.932 - -0.161	0.005
**Frequency of Prior Video**			
**Game Use** (Never)			
Daily Weekly Monthly Rarely	1.415 0.693 0.202 0.269	0.918–1.911 0.233–1.153 -0.398–0.802 -0.255–0.794	< 0.001 0.003 0.510 0.314
**Comfort Using VR** (0-100)	0.012	0.007–0.017	< 0.001
**Comfort with** **New Technology** (0-100)	0.012	0.005–0.019	< 0.001
**Comfort with** **Smart Phones** (0-100)	0.011	-0.004–0.025	0.141
**Comfort with** **a Computer** (0-100)	0.014	-0.002–0.030	0.085
**Comfort with** **a Television** (0-100)	0.016	0.002–0.030	0.029

For categorical variables reference group is presented in ( )

Multivariate analysis is found in Table 4. In the multivariate analysis age (-0.032 [-0.046–0.017], *p* < 0.001) and gender (-1.098 [-1.484 - -0.713], *p* < 0.001) continued to be the strongest predictors of score. Being a nurse was a positive predictor of a higher score when compared to physicians in the multivariate analysis, the opposite of the univariate analysis. Self-assessed comfort with VR remained a significant predictor, however, comfort with new technology became a negative predictor in the multivariate analysis. Comfort with other technologies such as smart phones, computers, and televisions were no longer predictive of score in the multivariate model.

**Table 4:** Multivariate analysis to identify predictors with higher scores

Multivariate Variables	Coefficient	95% CI	*p*-value
**Age** (years)	-0.032	-0.046 - -0.017	< 0.001
**Gender** (male)			
Female Non-binary	-1.098 0.151	-1.483 - -0.713 -1.244–0.1547	< 0.001 0.832
**Race** (Caucasian)			
Asian African American Other	0.111 -0.412 -0.268	-0.261–0.484 -0.910–0.086 -0.744–0.207	0.558 0.105 0.269
**Occupation** (Physician)			
Nurse Other Healthcare Sim Operations Other Non-Healthcare	0.549 0.015 0.148 0.035	0.004–1.093 -0.602–0.632 -0.383–0.678 -0.413–0.483	0.048 0.962 0.586 0.878
**Highest Degree**(Graduate)			
Undergraduate/HS	-0.352	-0.706–0.003	0.052
**Prior Use of VR** (Yes)			
No	-0.176	-0.515- 0.163	0.309
**Frequency of Prior Video**			
**Game Use** (Never)			
Daily Weekly Monthly Rarely	0.257 0.026 -0.385 -0.049	-0.294–0.807 -0.427–0.480 -0.985–0.215 -0.480–0.381	0.360 0.909 0.209 0.822
**Comfort Using VR** (0-100)	0.009	0.002–0.015	0.011
**Comfort with** **New Technology** (0-100)	-0.012	-0.020 - -0.003	0.008
**Comfort with** **Smart Phones** (0-100)	-0.007	-0.025–0.012	0.467
**Comfort with** **a Computer** (0-100)	0.013	-0.008–0.035	0.235
**Comfort with** **a Television** (0-100)	0.008	-0.005–0.021	0.234

For categorical variables reference group is presented in ( )

## Discussion

The utilization of VR for educational purposes is growing [4]. There is an imminent need to differentiate learners in virtual environments in order to prevent frustration and disengagement, both from situations when content is too easy or too challenging. This is especially true for VR where the learning experience may be their first experience with VR as a modality. As a comparator, assessments of reading skills (written material) or watching skills (video material) are not required given that in these spaces, learners are healthcare workers and have decades of experience with these modalities. High fidelity simulation might be similar in this regard. Although one would imagine the best predictor of success in a virtual environment would be prior experience in VR, 21 (47.7%) of our participants in the lowest scoring group had prior experience in VR. As such, participants may find themselves being overconfident in their first VR experiences which could lead to poor performance and may not result in success. Unfortunately, we do not have detailed information on the prior VR experience of participants, as we may have found a difference with novice versus experienced VR users in this study.

Our findings support some differentiators present in the literature. Gender has been reported in several works as a differentiator, with female participants scoring lower than male counterparts [11, 12]. One of the original arguments about the difference in performance in digital games such as VR between gender dealt with the idea that digital games and VR was seen mainly as a “male past time” with 62% of gamers reported as male in 2006 [13]. Over the last 15 years, this has evened out with only 55% of gamers identifying as males in 2021 [13]. In our sample, there was no difference in gender to video game exposure as a binary variable, however, male gamers were more likely to play games daily [17 (31.4%) vs. females 1 (2.4%) *p* = 0.006], but not weekly [15 (27.7%) vs. 11 (26.8%) *p* = 0.21]. Prior experience in VR was also no different between genders (Chi-Square: *p* = 0.207). The impact on prior gaming experience is discussed further below.

Another hypothesis centered around differing spatial perceptions between men and women, reporting that women have a higher incidence of “VR sickness” or motion sickness in virtual environments when compared with men [11, 14]. This has been further elucidated in studies examining differences in inter pupillary distance (IPD) and incidence of VR related motion sickness, citing that headsets may not have IPD settings optimized for women [11]. Newer headsets, such as the one used in this study, have IPD adjusters to address this issue. Likewise, system usability score (*p* = 0.530) as well as NASA-TLX scores (*p* = 0.462) were no different between genders. Finally, no participants in the study reported motion sickness after the experience, but this may vary depending on the content and length of the virtual experience.

Other factors not measured in this study may contribute to the gender disparity in virtual reality success. Identifying these factors is vital to allow a more equitable level of success in virtual reality educational materials. As the utilization of or potential reliance on virtual reality for medical education increases, it becomes even more vital that medical education sets all learners up for success.

Age was also identified as being inversely related to score, which has also been demonstrated in prior works. Xu et al. examined gameplay uncertainty, display type, and age on performance in exergames and found that middle aged adults performed worse than younger participants [15]. Age related declines in working memory, grip strength and muscle mass were cited as potential drivers. Although our experience was physical and required motion, it was likely a less vigorous experience than seen in the study by Xu et al. [15]. In our study, age and NASA-TLX physical demand scores were very weakly correlated but missed the significance threshold (Pearson Correlation Coefficient: 0.180, *p* = 0.08). Similarly, age distributions between those with and without prior VR experience were not different (Mann-Whitney U Test, *p* = 0.405). Our investigation did identify a difference in age distributions stratified by frequency of game use (Fig. 2, *p* < 0.001). Of 26 participants who reported never playing video games in the past, 14 (53.8%) reported the primary reason being that they did not exist at that time, supporting the prior data regarding age and video game experience.

Prior use of video games have also been shown to correlate with success in VR [16]. Playing video games improves spatial performance and cognition, an important skill for success in virtual environments. Our study confirms these prior findings with those playing video games daily or weekly having improved scores (Table 3). This difference was ablated in our multivariate analysis when controlling for age and gender. Although co-linearity statistical analysis was normal (Supplementary Table 1), it may still be that age, gender, and frequency of video game use are too related to be teased out in our dataset. Our sample size was too small to perform subgroup analysis when stratified by multiple parameters. Further work in this area is needed.

Our study demonstrated self-perceived comfort with VR and new technology were also predictive of success in VR. This supports the common notion that those who are “tech savvy” are more likely to be successful with new technologies such as virtual reality. These self-perceived factors may allow instructors to stratify VR participants by comfort with VR and new technology and focus orientation and training activities on those with low comfort. This can be done through utilization of tutorials or instruction manuals for the associated hardware or software.

Our study has several limitations. Although our sample was random, the study population are all individuals attending a simulation conference, making our results less generalizable to the general public. Additionally, because of the heterogeneity of our study population, our test experience was chosen to be a non-medical experience in order to allow varying levels of medical knowledge to not impact the study findings. Because of this, the use of a non-medical experience may limit the generalizability to a medical VR experience. Our test experience, although standardized, was a single experience, and as such our results could have been different if a different experience had been chosen. Another limitation includes the low incidence of video game use, which could have impacted our results because subjects needed to adapt to VR and video game use. This could have potentially inflated the impact of prior video game use on VR performance and may not apply to medical VR simulation. Self-selection bias may have also impacted our study, meaning subjects with less experience may have been less likely to volunteer to participate, which could have impacted our final results. It was also brief, and as such may underestimate the impact of motion or VR sickness that may be accompanied by longer experiences.

In conclusion, our study demonstrated that performance during an immersive VR experience was mostly correlated with age and gender. Self-perceived comfort with VR was also predictive of performance and may serve as a better screening question to stratify participants in VR experiences instead of a binary response to prior VR experience. Future studies evaluating this topic in medical simulation and in the general population are needed to further expand our understanding of VR adaptation.

### Electronic supplementary material

Below is the link to the electronic supplementary material.

Supplementary Material 1

## Data Availability

The datasets used and analyzed during the current study are available from the corresponding author on reasonable request.

## Abbreviations

VR: Virtual Reality
HFS: High-Fidelity Simulation
TEA: Technology Experience Area
NASA-TLX: NASA Task Load Index
VIF: Variance Inflation Factor
IPD: Inter pupillary distance
