# Supplementary material for: Relationship between demographic and social variables and performance in virtual reality among healthcare personnel: an observational study
Source: BMC Med Educ. 2024 Mar 4;24:227. doi: 10.1186/s12909-024-05180-0 (PMC10981290; doi:10.1186/s12909-024-05180-0)
Supplement: Supplementary file 1 — Supplementary Material 1 [file 12909_2024_5180_MOESM1_ESM.docx]

**Supplemental Table 1:** Co-linearity Statistics and Variance Inflation Factor (VIF)

| **Variable** | **Co-linearity Tolerance** | **VIF** |
| --- | --- | --- |
| Age | 0.833 | 1.2 |
| Gender | 0.657 | 1.52 |
| Race | 0.916 | 1.09 |
| Occupation | 0.734 | 1.36 |
| Highest Degree | 0.744 | 1.35 |
| Frequency Playing Video Games | 0.485 | 2.06 |
| Comfort with VR | 0.442 | 2.62 |
| Comfort with Using New Technology | 0.324 | 3.08 |
| Comfort with Smart Phones | 0.262 | 3.81 |
| Comfort with Computers | 0.260 | 3.84 |
| Comfort with a TV | 0.548 | 1.82 |
